# Long Noncoding RNAs and RNA-Binding Proteins in Oxidative Stress, Cellular Senescence, and Age-Related Diseases

**DOI:** 10.1155/2017/2062384

**Published:** 2017-07-25

**Authors:** Chongtae Kim, Donghee Kang, Eun Kyung Lee, Jae-Seon Lee

**Affiliations:** ^1^Department of Biochemistry, The Catholic University of Korea College of Medicine, Seoul 06591, Republic of Korea; ^2^Department of Molecular Medicine and Hypoxia-Related Disease Research Center, Inha University College of Medicine, Incheon 22212, Republic of Korea

## Abstract

Cellular senescence is a complex biological process that leads to irreversible cell-cycle arrest. Various extrinsic and intrinsic insults are associated with the onset of cellular senescence and frequently accompany genomic or epigenomic alterations. Cellular senescence is believed to contribute to tumor suppression, immune response, and tissue repair as well as aging and age-related diseases. Long noncoding RNAs (lncRNAs) are >200 nucleotides long, poorly conserved, and transcribed in a manner similar to that of mRNAs. They are tightly regulated during various cellular and physiological processes. Although many lncRNAs and their functional roles are still undescribed, the importance of lncRNAs in a variety of biological processes is widely recognized. RNA-binding proteins (RBPs) have a pivotal role in posttranscriptional regulation as well as in mRNA transport, storage, turnover, and translation. RBPs interact with mRNAs, other RBPs, and noncoding RNAs (ncRNAs) including lncRNAs, and they are involved in the regulation of a broad spectrum of cellular processes. Like other cell fate regulators, lncRNAs and RBPs, separately or cooperatively, are implicated in initiation and maintenance of cellular senescence, aging, and age-related diseases. Here, we review the current understanding of both lncRNAs and RBPs and their association with oxidative stress, senescence, and age-related diseases.

## 1. Introduction

**Figure 1:**
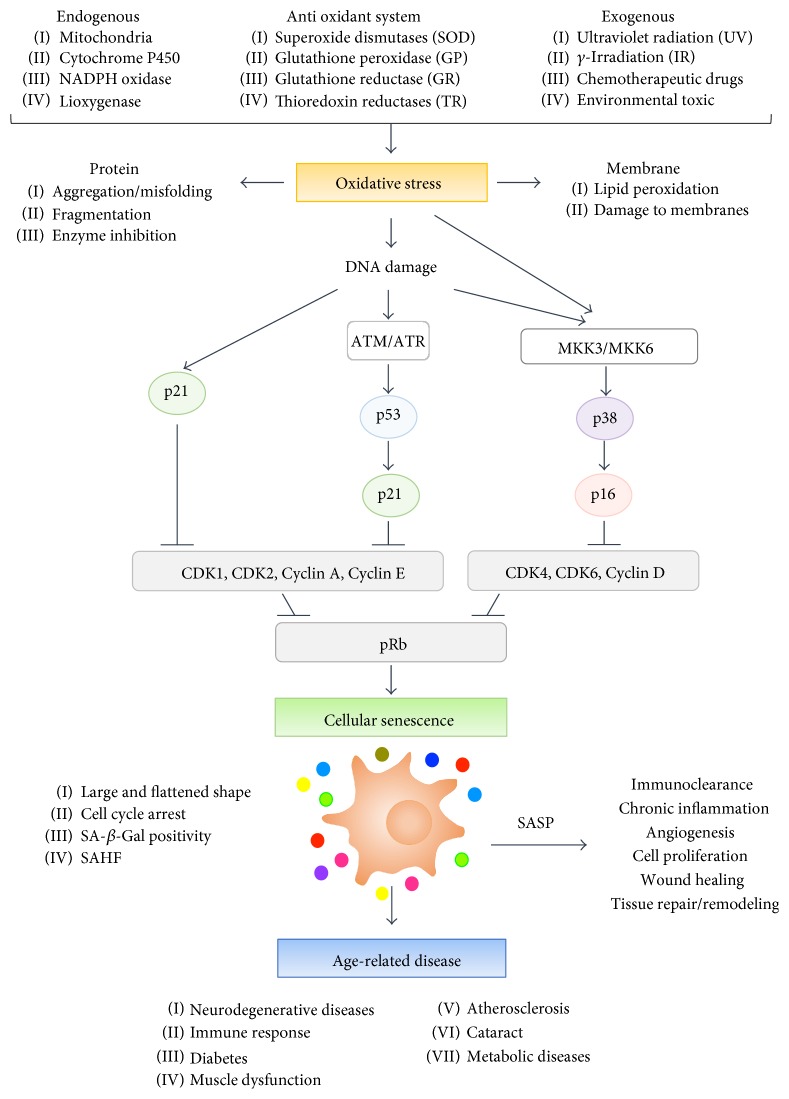
Schematic relationships among DNA damage and oxidative stress, cellular senescence, and age-related diseases.

Cellular senescence is a biological process in which cells cease growth permanently. Hayflick and Moorehead firstly described replicative senescence (RS), which is an exhaustion of replicative potential in human diploid fibroblasts after continuous cultivation [1, 2]. More than a half-century from that first description that early concept of cellular senescence has been remarkably extended in recent days. RS can be considered a defense mechanism that limits proliferation potential of older cells containing irreparable and dangerous mutations. In contrast to RS, which is driven by telomere shortening, cells can prematurely undergo senescence in response to diverse forms of cellular stresses. Stress-induced cellular senescence (SIPS) can be triggered by DNA damage, oncogenic mutations, strong mitotic signals, genomic instability, lack of nutrients, improper cell contacts, and many other factors [3]. An excess of reactive oxygen species (ROS) specifically participates in induction and maintenance of cellular senescence. ROS including superoxide anion, hydrogen peroxide, and hydroxyl radicals are inevitably generated as byproducts of aerobic metabolism and are also derived from radiation, chemotherapeutic agents, carcinogens, and other intrinsic and extrinsic factors. Physiological ROS level regulates signal transduction, gene expression, and proliferation. However, ROS shifts from physiological to pathophysiological level are referred to as oxidative stress. Oxidative stress results in damage to lipids, proteins, and nuclear and mitochondrial DNA and is involved in various changes, such as epigenetic modification and signaling pathways, finally resulting in cellular senescence [4]. Cellular senescence programs are induced by persistent activation of the p53/p21 stress response pathway and/or the RB/p16 tumor suppressor pathway. Senescent cells are characterized by a variety of phenotypes including enlarged and flattened morphology, senescence-associated *β*-galactosidase (SA-*β*-Gal) activity, formation of senescence-associated heterochromatin foci (SAHF), and altered gene expression and protein processing [5]. In the last decade, many research groups have demonstrated that anticancer drugs and ionizing radiation can effectively induce SIPS in cancer cells in vitro and in vivo [6]. Currently, in accord with the role of senescence-associated secretory phenotypes (SASP) in tumor prevention, therapy-induced senescence is considered a powerful strategy for cancer treatment [7]. Although senescent cells irreversibly lose their dividing capability, they are metabolically active and secrete a myriad of SASP-related factors including cytokines, chemokines, growth factors, and proteases [1]. SASP can influence multiple facets of tissue microenvironments and contribute to the inflammatory response and many other aging phenotypes. Since cellular senescence can potentially contribute to various physiological and pathological aging processes, cellular senescence is an important hallmark of human aging and an attractive target for therapeutic exploitation [6–10]. Aging is the gradual deterioration of the physical, mental, and biological state of an organism with time, eventually resulting in increased vulnerability to death. Moreover, aging is the irreversible loss of physiological integrity and the major risk factor in various age-related diseases such as neurodegenerative diseases, immune response, metabolic diseases, muscle dysfunction, atherosclerosis, and cataract. Various factors and processes have been implicated in the initiation, regulation, and progression of the aging process. An “oxidative stress theory of aging” was proposed long ago, and oxidative stress is primarily associated with cellular senescence and aging. Several studies have shown that transcriptional events are implicated in the regulation of gene expression during oxidative stress responses, cellular senescence, and the pathogenesis of age-related diseases [11–14]. Activation of transcriptional factors such as p53, NF-*κ*B, HIF-1*α*, CEBP, STAT, and E2F1 governs mRNA expression via promoter activation, microRNA induction, and epigenetic regulation in senescence, aging, and age-related disease [11]. Schematic relationships among DNA damage and oxidative stress, cellular senescence, and age-related diseases are shown in Figure 1. Herein, we revisit current knowledge of the mechanistic, functional, and pathological roles of long noncoding RNAs (lncRNAs) and RNA-binding proteins (RBPs) that are primarily related to DNA damage, oxidative stress, cellular senescence, aging, and age-related diseases. In this review, we will not describe lncRNAs and RBPs associated with telomeres and cancer because those topics have already been extensively introduced in other reviews [15–18].

## 2. Long Noncoding RNAs

The lncRNAs are transcripts more than 200 nucleotide long that have no protein-coding potential. Moreover, they are poorly conserved, transcribed from the intergenic and intronic regions of genome primarily by polymerase II, 5′ methyl-capped, and polyadenylated in manner similar to that of mRNAs [16]. The lncRNAs modulate gene expression at all regulation levels: transcriptional, posttranscriptional, translational, and posttranslation. They can regulate gene expression via interaction with chromatin modifiers, RBPs, DNA, and RNA [15]. To date, many lncRNAs have been characterized. Most of those are nuclear localized and act as enhancer RNAs (eRNAs), chromatin modifiers via recruitment of various DNA methyltransferases, and histone modifiers via Polycomb repressive complexes or histone methyltransferases [16]. Some lncRNAs are transported to the cytoplasm and regulate translation or mRNA stability. Moreover, lncRNAs affect key cellular processes such as proliferation, differentiation, quiescence, senescence, stress and immune response, and many other cellular functions related to the biology of aging [19].

### 2.1. DNA Damage Response and Oxidative Stress

#### 2.1.1. LincRNA-p21

*LincRNA-p21* is 3.1 kb long and is transcribed from the opposite strand to p21 (CDKN1A) in a p53-dependent manner [20]. *LincRNA-p21*, which is also induced by hypoxia and/or hypoxia inducible factor-1*α* (HIF-1*α*), is able to bind HIF-1*α* and VHL, and it disrupts the VHL–HIF-1*α* interaction. This disassociation attenuates VHL-mediated HIF-1*α* ubiquitination and causes HIF-1*α* accumulation. These results indicate a positive feedback loop between HIF-1*α* and *lincRNA-p21* under hypoxia [21]. In addition, *LincRNA-p21* is highly inducible by UVB through a p53-dependent pathway and plays key role in the UVB-induced apoptotic pathway [22].

#### 2.1.2. LincRNA-RoR

*LincRNA-RoR*, a 2.6 kb long transcript, was first described as having potentially important functions in embryonic stem cells and induced pluripotent stem cells (iPSCs) [19]. *LincRNA-RoR* regulates genes involved in the p53 response, such as responses to oxidative stress and DNA damage [23]. Depletion of p53 can partially rescue the apoptotic phenotype by ablation of *lincRNA-RoR*. *LincRNA-RoR* dramatically represses DNA damage-induced p53 compared to that in unstressed cells. Depletion of *lincRNA-RoR* did not regulate *p53* mRNA levels, suggesting posttranscriptional regulation of p53. Mechanistically, *lincRNA-RoR* has a 28-base heterogeneous nuclear ribonucleoprotein I- (hnRNP I-) binding motif and directly interacts with phosphorylated hnRNP I in the cytoplasm. The interaction between *lincRNA-RoR* and phosphorylated hnRNP I directly represses p53 translation and results in the modulation of cell-cycle progression and apoptosis. Thus, *lincRNA-RoR* and p53 act within an autoregulatory feedback loop in response to cellular stress [24]. A recent study revealed that *lincRNA-RoR* can epigenetically regulate the expression of *TESC* by recruiting G9A methyltransferase in the *TESC* promoter [25].

#### 2.1.3. Pint

*Pint* (p53-induced noncoding transcript), previously named *lincRNA-Mkln1*, has highly conserved canonical p53-binding motifs in the promoter and is a transcriptional target of p53 [20]. *Pint* is a nuclear lincRNA and is transcribed from an intergenic region on mouse chromosome 6. *Pint* has three p53 response elements and is directly regulated by p53 upon DNA damage [26]. Depletion of *Pint* significantly decreases cell proliferation, and overexpression of *Pint* conversely increases cell growth. *Pint* directly interacts with Polycomb repressive complex 2 (PRC2) and represses expression of PRC2 targeting genes via H3K27 trimethylation. *PINT*, the *pint* human ortholog, is also regulated by p53. However, overexpressed *PINT* diminishes tumor cell proliferation, indicating both analogy and difference between murine *Pint* and human ortholog *PINT* [26].

#### 2.1.4. PANDA

To detect functional noncoding RNAs (ncRNAs) in the regulatory region of human cell-cycle genes, ultrahigh-density array fabrication was performed and the lncRNA *PANDA* (P21-associated ncRNA DNA damage-activated) was identified at the *CDKN1A* locus [27]. *PANDA* is specifically induced by DNA damage in a p53-dependent manner. *PANDA* is a 5′-capped, polyadenylated lncRNA located approximately 4.5 kb upstream of the *CDKN1A* transcriptional start site. In human fibroblasts treated with doxorubicin, *PANDA* prevents NF-YA activation, through its association with NF-YA, finally suppressing transcription of proapoptotic genes. Thus, *PANDA* induced by DNA damage impedes apoptosis through recruitment of NF-YA [27]. Another study reported that *PANDA* can stabilize p53 proteins in response to DNA damage [28]. In addition, it was revealed that silencing of *PANDA* causes G1 arrest via an increase in the mRNA level of cyclin-dependent kinase inhibitor p18 [29].

#### 2.1.5. LncRNA-JADE

*LncRNA-JADE* is induced by ATM-NF-*к*B signaling and is mainly localized in the nucleus in the DNA damage response (DDR) [30]. In response to DDR, increased *lncRNA-JADE* interacts with breast cancer type 1 susceptibility protein (Brca1) and induces expression of Jade1, a major component of human acetylase binding to ORC 1 (HBO1) histone acetylation complex. Consequently, depletion of *lncRNA-JADE* renders sensitivity to DNA damaging drugs through the functional link between DDR and histone H4 acetylation in the DDR. [30].

#### 2.1.6. H19

*H19* was first described as an imprinted ncRNA transcript at the *Igf2* locus. A number of studies have reported that *H19* is upregulated in both primary and metastatic tumors and is closely involved in migration, angiogenesis, and inflammatory diseases [31]. HIF-1*α* and p53 are involved in the upregulation of *H19* in hypoxic cancer cells [32]. Recently, it was demonstrated that *H19* expression is elevated under hypoxic conditions in mesenchymal stem cells [33]. In addition, overexpression of *H19* in diabetic rats can attenuate oxidative stress, inflammation, and apoptosis [34].

#### 2.1.7. ANRIL

*ANRIL* (antisense noncoding RNA in the INK locus) is transcribed in the antisense direction to the INK4B-ARF-INK4A locus and is transcriptionally upregulated by the transcription factor E2F1 in an ATM-dependent manner after DNA damage. Such elevated levels of *ANRIL* suppress the expression of INK4A-ARF-INK4B in the late-DDR stage, allowing negative feedback to the DDR. Thus, *ANRIL* helps the cell to return to a normal status at the completion of DNA repair [35].

#### 2.1.8. LncRNA-LET

*LncRNA-LET* (lncRNA low expression in tumor) transcripts are underexpressed in tumor tissues compared to their expression in paired nontumor tissues [36]. Moreover, *LncRNA-LET* is downregulated by hypoxia-induced histone deacetylase 3 (HDAC3) under hypoxic conditions. *LncRNA-LET* is associated with degradation of nuclear factor of activated T cells 90 kDa (NF90) protein via the regulation of ubiquitin-proteasome pathway. Since NF90 stabilizes *HIF-1α* mRNA without altering HIF-1*α* transcriptional activity, *lncRNA-LET* finally decreases HIF-1*α* stability due to its association with NF90. These findings illustrate that *lncRNA-LET* could be a key regulator of hypoxia signaling [36].

#### 2.1.9. LINK-A

*LINK-A* (long intergenic noncoding RNA for kinase activation) is 1.5 kb long and mainly localized in the cytoplasm [37]. *LINK-A* facilitates breast tumor kinase (BRK) activation through the recruitment of BRK to the EGFR : GPNMB heterodimeric complex upon HB-EGF stimulation. Consequently, activated BRK induces phosphorylation of HIF-1*α* at Tyr565 and inhibits hydroxylation of HIF-1*α*, finally resulting in HIF-1*α* stabilization. In addition, *LINK-A* interacts with leucine-rich repeat kinase 2 (LRRK2) and enhances phosphorylation of HIF-1*α* at Ser797. Phosphorylation of Ser797 increases transcriptional activation of HIF-1*α* via HIF-1*α*–p300 interaction. These events illustrate the magnitude and diversity of cytoplasmic lncRNA *LINK-A* in signal transduction related to HIF-1*α* under normoxic conditions [37].

### 2.2. Cellular Senescence and Aging

#### 2.2.1. 7SL

*7SL* is a 300 bp long transcript and an RNA component of signal recognition proteins (SRP) [38]. *7SL* is widely upregulated in cancer tissues and involved in cell proliferation. *7SL* decreases p53 translation and accumulation by interacting with the 3′-untranslated region (3′-UTR) of *TP53* mRNA, which encodes tumor suppressor p53. Depletion of *7SL* increases the occupancy of HuR to *TP53* mRNA and p53 production. *7SL*-depleted cells undergo cellular senescence and autophagy, indicating that *7SL* promotes cell growth via p53 suppression [39].

#### 2.2.2. HOTAIR

*HOTAIR* (HOX antisense intergenic RNA) was first identified as HOX lncRNA located in the HOXC locus through transcriptomic analyses of HOX loci [40]. This antisense lncRNA increases the occupancy of Suz12 on the HOXD locus and silences HOXD locus genes by changing the chromatin structure [40]. *HOTAIR* enhances cancer progression and malignancy by leading to altered H3K27 methylation due to retargeting of PRC2 [41]. Depletion of *HOTAIR* induces cell-cycle arrest in various cancer types. In addition, *HOTAIR* can contribute to cellular senescence via a positive feedback loop cascade of an NF-*к*B–HOTAIR axis [42].

#### 2.2.3. UCA1

*UCA1* (urothelial carcinoma-associated 1), an lncRNA with a length of 1.4 kb, was first identified in bladder cell carcinoma [43]. *UCA1*, a direct target of coactivator of AP1 and estrogen receptor *α* (CAPER*α*)/T-box3 (TBX3) repression, sequesters hnRNP I, which suppresses transcription of *CDKN2A* and destabilizes *CDKN2A* mRNA [44]. Oncogenic stress dissociates the CAPER*α*/TBX3 corepressor and activates *UCA1*. CAPER*α*/TBX3 and UCA1 coordinately induce oncogene-induced senescence (OIS). In addition, *UCA1* can bind with hnRNP I and competitively inhibit hnRNP I binding to *p27* mRNA [45]. hnRNP I enhances translation of *p27* mRNA, and there is a negative correlation between p27 expression and *UCA1* level.

#### 2.2.4. LincRNA-p21

Overexpressed *lincRNA-p21* increases p21 expression at both mRNA and protein levels, and it impedes cell-cycle progression [46]. *LincRNA-p21* is necessary for the recruitment of hnRNP K to the p53 response element and for increasing the binding efficiency of p53 on the p21 promoter region. Moreover, *lincRNA-p21* affects the G1/S checkpoint and p21 levels through deregulated expression and altered chromatin state of some Polycomb target genes. Thus, *lincRNA-p21* is required for the positive regulation of p21 expression and finally is involved in cellular senescence [46].

#### 2.2.5. ANRIL

*ANRIL* is a 3.8 kb long lncRNA transcribed in an antisense orientation from the INK4B/ARF/INK4A gene cluster, and it overlaps with the promoter of p14/ARF and the two exons of p15/CDKN2B [47]. *ANRIL* is required for the recruitment of chromobox (CBX7), a component of PRC1, to the INK4B/ARF/p16 gene locus. This complex exhibits high-affinity binding to methylated histone H3 at lysine 27 (H3K27me) and represses the transcription of INK4b/ARF/INK4a [48]. Moreover, depletion of *ANRIL* disrupts the binding of suppressor of *zeste* 12 protein homolog (Suz12), a component of PRC2, to INK4B locus, and increases the expression of p15 [49, 50]. Recent studies have reported that *ANRIL* promotes silencing of KLF2 and P21 transcription via epigenetic silencing [51]. Such epigenetic transcriptional repression of INK4B/ARF/INK4A by *ANRIL*, which is associated with senescence, was reviewed by Aguilo et al. [52].

#### 2.2.6. ANRASSF1

*ANRASSF1* (antisense intronic noncoding *RASSF1*) is an intronic lncRNA transcribed from the antisense to RAS-association domain family member 1A (*RASSF1A*) gene [53]. *RASSF1A*, a tumor suppressor gene, is associated with cell-cycle arrest and senescence via p53-independent regulation of p21 [54]. Highly expressed *ANRASSF1* recruits PRC2 to the *RASSF1A* promoter and increases the H3K27me3 level, resulting in decreased *RASSF1A* expression. Therefore, *ANRASSF1* mediates cellular senescence through the epigenetic inactivation of the *RASSF1A* gene [53].

#### 2.2.7. PANDA

*PANDA* (P21 associated ncRNA DNA damage-activated) is capable of interacting with scaffold-attachment-factor A (SAFA) [55]. SAFA is a nuclear protein that is able to bind DNA and RNA, including ncRNA, and is involved in transcriptional and posttranscriptional regulation by acting as an adaptor molecule for DNA-RNA-protein interactions [56]. In proliferating cells, the SAFA and *PANDA* interaction recruits Polycomb repressive complex 1 (PRC1) and PRC2 complexes to senescence target genes including *CDKN1A* in order to silence their expression [55]. Thus, *PANDA* depletion leads to senescence phenotypes by derepression of p21 due to disruption of SAFA-PANDA-PRC interactions. However, in senescent cells, *PANDA* sequesters transcription factor NF-YA and limits the expression of NF-YA-E2F-coregulated proliferation-promoting genes. Therefore, *PANDA* levels modulate cell fates to enter or exit from senescence [55].

#### 2.2.8. FAL1

*FAL1* (focally amplified lncRNA on chromosome 1) was identified from a genome-wide analysis of somatic copy number alterations [57]. *FAL1* interacts with epigenetic repressor BMI1 protein, a subcomponent of PRC1, and increases BMI1 stability. Thus, *FAL1* can negatively regulate a large number of genes such as *CDKN1A*, *FAS*, and *BTG2*. In addition, *FAL1* promotes tumor proliferation and represses senescence primarily by decreasing *CDKN1A* transcription [58].

#### 2.2.9. MIR31HG

Whereas *MIR31HG* lncRNA is upregulated during OIS, its depletion promotes p16-dependent senescence phenotypes [59]. *MIR31HG* interacts with the INK4A and *MIR31HG* genomic loci and mediates repression of the INK4A locus with Polycomb group (PcG) proteins. *MIR31HG* plays a role during OIS as a transcriptional regulator of p16 via direct interaction with PcG proteins [59].

#### 2.2.10. SALNR

*SALNR* (senescence-associated long noncoding RNA) expression is downregulated in senescent human fibroblasts. *SALNR* interacts with NF90, a RNA-binding protein involved in microRNA (miRNA) biogenesis, and regulates its nuclear localization. *SALNR* and the NF90 complexes impede premature senescence through the regulation of senescence-associated miRNAs, specifically miR-181a and miR-22 [60].

#### 2.2.11. VAD

*VAD* is a vlincRNA (very long intergenic ncRNA) that is differentially expressed in RAF-induced senescence and is localized in the chromatin. *VAD* is involved in the maintenance of senescence features. Mechanistically, *VAD* modulates chromatin structure in *cis* and increases the expression of INK4 genes in *trans*. *VAD* decreases the occupancy of the repressive histone variant H2A.Z at *INK*4 promoters during senescence induction [61].

### 2.3. Age-Related Diseases

#### 2.3.1. Neurodegenerative Diseases

*(1) Alzheimer's Disease*. Alzheimer's disease (AD) is the most common neurodegenerative disease and accounts for the majority of dementia cases. Amyloid *β* (A*β*) plaques and neurofibrillary tangles are the two primary pathological hallmarks of AD. The amyloid cascade hypothesis suggests that deposition of A*β* might be cause of neuronal dysfunction and death of brain tissue in AD. Recently, the cleavage patterns of amyloid precursor protein (APP) to A*β* peptides (A*β*_1–40_ and A*β*_1–42_) by secretases, small oligomers of A*β* (2~12 peptides), A*β* concentration, and A*β* stability have been proposed as important factors in AD [62].

*BC200* is a 200 bp long RNA pol III-transcribed lncRNA that is predominantly expressed in the brain [63]. *BC200* is downregulated in normal aged brains, but *BC200* is significantly upregulated in AD brains. Specifically, *BC200* is highly expressed in AD-related regions (e.g., Broadmann's area 9) compared to its expression in nonrelated regions (e.g., area 17) [64].

*BACE1-AS* (BACE1-antisense transcript) is a 2 kb long transcript from the antisense strand of *β*-secretase-1 (*BACE1*) and is a crucial enzyme in AD pathology. *BACE1-AS* regulates *BACE1* mRNA and protein expression in vitro and in vivo. In response to cell stress, elevated *BACE1-AS* increases *BACE1* mRNA and protein levels due to RNA duplex formation, generating additional A*β* 1–42 peptides [65]. Moreover, modulation of *BACE1* and the *BACE1-AS* transcript can participate in the alteration of oligomeric A*β* aggregation pattern and A*β*-related hippocampal neurogenesis [66].

*NDM29* (neuroblastoma differentiation marker 29) is a cytoplasmic lncRNA transcribed by polymerase (pol) III. *NDM29* is highly expressed in neuroblastoma cells and is involved in neuroblastoma maturation [67]. In addition, elevated *NDM29* expression is detected in the brain of AD patients. *NDM29*-dependent cell maturation induces APP synthesis and results in an increase of A*β* secretion. Moreover, an increase in the production of copies of *NDM29* transcripts can be driven by inflammatory stimuli [68].

*17A* is a 159 bp long lncRNA synthesized by RNA pol III that induces an increase of GABA B2 receptor splice variant B, which affects GABA-B function. Thus, *17A* impairs GABA-B signaling and might enhance A*β* secretion. In addition, *17A* is upregulated in AD compared to its level in control tissues [69]. Other lncRNAs such as *51A*, *NAT-Rad18*, and *GNDFOS* might also be involved in AD [70, 71].

*(2) Parkinson's Disease*. Parkinson's disease (PD) is a common and complex neurodegenerative disease characterized by defects in the body's motor functions (slow or lack of movements, and tremor). The classical features of PD are associated with Lewy bodies and loss of dopaminergic neurons in the substantia nigra pars compacta in the midbrain. The resultant deficiency of dopamine ultimately induces movement disorder, which is a characteristic of PD [72].

*AS Uchl1* (antisense to mouse ubiquitin carboxy-terminal hydrolase L1) is a 1.2 kb lncRNA transcribed from the opposite strand of the ubiquitin carboxy-terminal hydrolase L1 (*Uchl1*) gene and induces *UChl1* translation. *AS Uchl1* is expressed in mesencephalic regions, which are degenerated in PD. *AS Uchl1* is regulated by Nur11, a major transcription factor functioning in the differentiation and maintenance of dopaminergic neurons. Expressions of *AS Uchl1* and UCHL1 have been decreased in PD models in vitro and in vivo [73].

*NaPINK1* is transcribed from the antisense direction of the *PINK1* gene, which is implicated in PD through an association with unbalanced mitochondrial homeostasis. As *naPINK1* is able to stabilize PINK1 splice variant (svPINK1) expression in neurons via a dsRNA-mediated mechanism, *naPINK1* might be involved in PD through regulation of the *PINK1* locus [71, 74].

*(3) Huntington's Disease*. Huntington's disease (HD) is a dominantly inherited disease characterized by chorea, psychiatric problems, and dementia. HD is caused by mutation in the *huntingtin* (*HTT*) gene. Expansion of the CAG-triplet repeat sequence within the first exon of *HTT* results in abnormal protein production, which gradually leads to death of brain cells [75].

*TUG1* (taurine upregulated gene 1) was first identified in a screen for genes upregulated by taurine in developing retinal cells [76] and, subsequently, in a genome analysis to examine lncRNAs physically associated with chromatin-modifying complexes [77]. Depletion of *TUG1* increases the phenotypes of apoptosis [76], and *TUG1* expression is elevated in HD [78]. Mechanistically, *TUG1*, a direct transcriptional target of p53, combines with enhancer of *zeste* homolog 2 (EZH2), a component of the PRC2 complex, and epigenetically regulates gene expression. *TUG1* and EZH2 bind to the promoter of homeobox B2 (*HOXB7*) and represses *HOXB7* expression [79].

*MEG3* is highly expressed in adult human and mouse brains and is differentially expressed in HD patients. *MEG3* can associate with the PRC2 complex and is found in the chromatin region in the nucleus, suggesting that *MEG3* might be involved in epigenetic regulation in HD [16, 78]. Other lncRNAs such as *HAR1* (human accelerated region 1), *NEAT1* (nuclear paraspeckle assembly transcript 1), and *DGCR5* (DiGeorge syndrome critical region gene 5) also exhibit altered expression in HD patients, as shown by microarray studies [16, 78].

*HTTAS* (huntingtin antisense) is a natural antisense transcript at the HD CAG repeat. *HTTAS* is mainly spliced into *HTTAS-V1* (exons 1 and 3) and *HTTAS-V2* (exons 2 and 3). *HTTAS-V1* expression is reduced in human HD frontal cortex, and its overexpression negatively regulates HTT transcription [80].

#### 2.3.2. Immune Response

The immune response is a wide variety of physiological and pathological processes originating from immune system activation. It is triggered by pathogens, antigens, tissues injury, and other noxious stimulations. The innate immune response provides immediate defense against infection and is evolutionarily conserved. The adaptive immune response is highly specific to particular pathogens and provides long-lasting protection. Adaptive immune responses are mainly mediated antibody and cell-mediated immune responses. The inflammatory response is considered an innate immune response. Inflammation functions to eliminate the initial cause of the original insult and to initiate tissue repair, which are regulated by immune mediators including cytokines, chemokines, and soluble inflammatory proteins [81].

*THRIL* (TNF*α*- and hnRNP L-related immunoregulatory lincRNA) is an approximately 2 kb lncRNA that changes expression upon activation of innate immune signaling in macrophages. *THRIL* recruits heterogeneous nuclear ribonucleoprotein L (hnRNP L) to the TNF*α* promoter and increases the secretion of TNF*α*, an inflammatory cytokine. An increase in TNF*α* downregulates *THRIL* expression via a negative feedback mechanism. Moreover, *THRIL* is associated with maintaining expression of many innate immunity-associated genes [82].

*Lnc-DC* is exclusively expressed in human conventional dendritic cells and is involved in dendritic cell (DC) differentiation and DC capacity to stimulate T cell activation. *Lnc-DC* prevents signal transducer and activator of transcription 3 (STAT3) dephosphorylation by SHP1 through a direct association with STAT3. *Lnc-DC* is known as a specific regulator of DC differentiation and function [83].

*Lnc-IL7R*, which overlaps with the 3′-UTR of interleukin-7 receptor *α* (*IL7R*) gene, shows altered expression in response to LPS stimulation. *Lnc-IL7R* functionally diminishes the LPS-induced inflammatory response and is mechanistically involved in trimethylation of H3K27 at the *E-selectin* and *VCAM-1* promoters. *Lnc-IL7R* is a regulator of proinflammatory genes via epigenetic modification [84].

*LincRNA-EPS* is downregulated in response to inflammatory triggers. Gain-of-function and rescue studies have revealed that *lincRNA-EPS* represses transcriptions of immune response genes by interacting with hnRNP L. *LincRNA-EPS* has a critical role in restraining lethal inflammatory responses [85].

#### 2.3.3. Diabetes

Diabetes is a metabolic disease associated with high blood sugar levels. Diabetes can be caused by the pancreas not producing insulin (type 1 diabetes (T1DM)) or by insulin resistance (type 2 diabetes (T2DM)). The majority of type 1 diabetes cases are attributed to a T cell-mediated autoimmune attack, which leads to loss of the insulin-producing beta cells of the islets of Langerhans in the pancreas. It is traditionally termed juvenile diabetes and is partially inherited. T2DM is the most common type of diabetes. Insulin resistance in T2DM might be combined with reduced insulin secretion and defective responsiveness of insulin receptors [86].

*RNCR3* is involved in diabetes-induced retinal neurodegeneration [87]. Knockdown of *RNRC3* reduces the release of cytokines and results in fewer apoptotic retinal cells and improved visual function. *RNCR3* increases in response to high glucose stress in vitro and in vivo and regulates retinal endothelial cell function through the RNCR3/KLF2/miR-185-5p network [88].

*MEG3* is reduced in the retinas of STZ-induced diabetic mice and in endothelial cells under high glucose and oxidative stress. *MEG3* knockdown aggravates diabetes-related retinal vessel dysfunction, which is mainly mediated by activation of PI3K/AKT signaling [89]. *MEG3* expression is upregulated in hepatocytes through histone acetylation in high-fat diet and ob/ob mice. In addition, *MEG3* is involved in hepatic insulin resistance via an increase in FoxO1 expression [90].

*HI-LNC25* was first identified in a transcriptome mapping study of human pancreatic islets and *β* cells [87]. *HI-LNC25* is a *β* cell-specific lncRNA and an integral component of *β* cell differentiation and maturation [87]. Depletion of *HI-LNC25* decreases expression of GLIS3 mRNA, which is associated with pancreatic *β* cell function and mass maintenance [91]. *KCNQ1OT1* and *HI-LNC45*, which were previously genetically associated with T2DM [92], are significantly dysregulated in diabetes islets [91].

#### 2.3.4. Muscle Dysfunction

Muscle development is a multistep process that includes myogenesis, muscle differentiation, and regeneration. Myogenesis is a tightly regulated developmental program to direct myoblasts to form muscle fibers. Myogenic pathways are primarily governed by transcription factors, MyoD, Myf5, myogenin, and MRF4 at the molecular level. Impairment of these processes might be a cause of muscle dysfunction and is an age-related pathological phenomenon [93].

*SRA* (steroid receptor RNA activator) was initially characterized as an lncRNA functioning to enhance steroid receptor-dependent gene expression [94]. *SRA* has an unusual property that functions as both *SRA* RNA and SRAP protein through alternative splicing. The ratio between *SRA* RNA and SRAP increases during myogenic differentiation, but there is no increase in myotonic dystrophy patients. *SRA* RNA is an enhancer of myogenic differentiation and myogenic conversion through regulation of MyoD activity [95].

*MUNC* (*MyoD* upstream noncoding RNA) is transcribed from the upstream of myogenic differentiation (MyoD), a master transcriptional regulatory factor in muscle differentiation and specifically expressed in skeletal muscle. *MUNC* depletion reduces myoblast differentiation and impairs muscle regeneration in vivo. *MUNC* is involved in gene expressions of *MyoD*, *Myogenin*, and *Myh3* (myosin heavy chain) by acting in *trans. MUNC* also stimulates the transcription of other genes that are not recognized as MyoD-inducible genes. *MUNC* is an evolutionarily conserved promyogenic lncRNA that acts directly or indirectly on multiple promoters to increase myogenic gene expression [96].

*Linc-RAM* (linc RNA activator of myogenesis) is a skeletal muscle-specific lncRNA that localizes in both cytoplasm and nucleus of myoblast. Depletion of *linc-RAM* impairs myoblast differentiation and muscle regeneration. Mechanistically, *linc-RAM* promotes assembly of the MyoD-Baf60c-Brg1 complex and facilitates the recruitment of the SWI/SNF core on target myogenic genes, resulting in transcription of myogenic differentiation genes [97].

Other muscle-specific lncRNAs such as *linc-MD1* and *lncRNA Dum* are also involved in the control of muscle gene expression and muscle regeneration [98, 99].

#### 2.3.5. Atherosclerosis

Atherosclerosis is the primary cause of heart disease and stroke. It is a chronic disease of the large arteries and is characterized by narrowing or closing of an artery with lipids and fibrous elements. Pathological studies have provided evidence of the critical role of endothelium in mediating inflammation and accumulation of oxidized low-density lipoproteins (LDL) in the intima to recruit monocytes and form macrophage-derived foam cells [100].

*SENCR* (smooth muscle and endothelial cell-enriched migration/differentiation-associated long noncoding RNA) is an antisense transcript from the first intron of *friend leukemia virus integration 1* (*FLI1*) and is localized in the cytoplasm. *SENCR* is highly expressed in both smooth muscle and endothelial cells [101]. *SENCR* impedes migration and proliferation of smooth muscle cells through the regulation of FoxO1 and TRPC6 expression [102]. In addition, *SENCR* is associated with the regulation of endothelial cell differentiation and angiogenic capacity of human umbilical endothelial cells (HUVECs) [103].

Recent studies have reported several lncRNAs that are involved in atherosclerosis-related smooth muscle cell, endothelial cell, macrophage, and lipid metabolism regulation, suggesting a potential function of such lncRNAs in atherosclerosis development [104].

#### 2.3.6. Cataract

Cataract is characterized by the clouding of an eye's lens. Cataract accounts for half the cases of blindness. Lens proteins denature and degrade over time, and this process is accelerated by age and diseases such as diabetes and hypertension. ROS may be mechanistically involved in cataractogenesis. The only treatment for cataract is surgery in the current state of technology [105].

*LncRNA-MIAT* (lncRNA myocardial infarction associated transcript) is highly expressed in patients with cataracts and is involved in the maintenance of human lens epithelial cells (HLECs) whose dysfunction results in cataract formation. *MIAT* regulates viability, proliferation, and migration of human HLECs in response to oxidative stress. Mechanistically, *MIAT* acts as a competing endogenous RNA (ceRNA) and can regulate HLEC function through a feedback loop with AKT and miR-150-5p [106].

**Figure 2:**
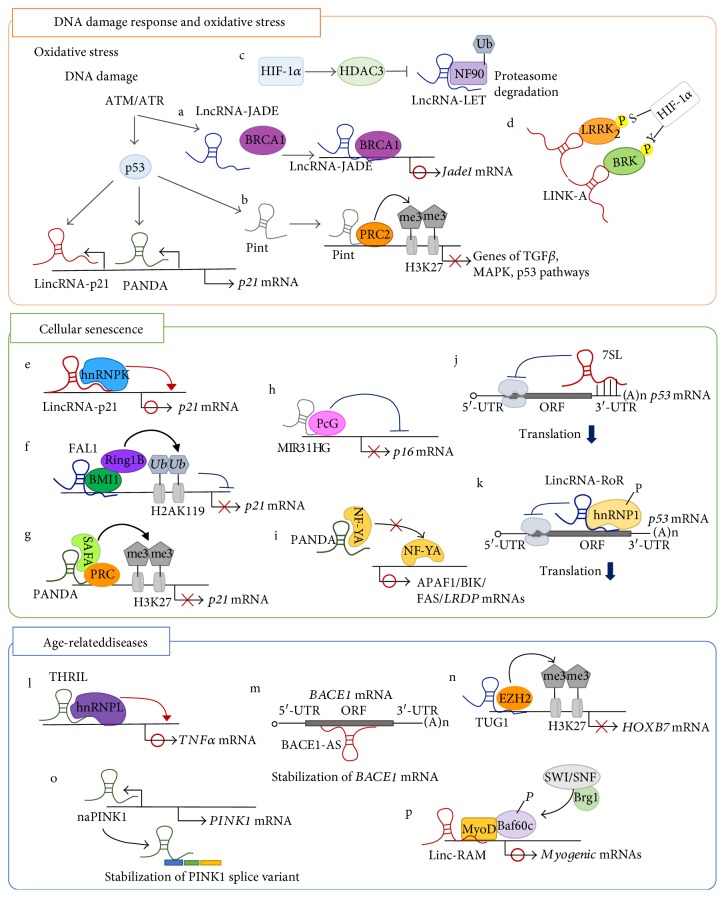
A mechanistic diagram of representative IncRNAs involved in DNA damage and oxidative stress, cellular senescence, and related diseases.

A mechanistic diagram of representative lncRNAs involved in DNA damage and oxidative stress, cellular senescence, and age-related diseases is shown in Figure 2.

## 3. RNA-Binding Proteins

The RBPs have a pivotal role in mediating posttranscriptional regulation of gene expression by affecting pre-mRNA splicing and maturation as well as mRNA transport, storage, turnover, and translation [107]. RBPs can regulate a broad spectrum of cellular process including cell proliferation, death, differentiation, and development, and differential expression or altered activity of certain RBPs is involved in the pathogenesis of several human diseases [107–109]. RBPs interact with mRNAs via a limited set of modular RNA-binding domains (RBDs), such as the heterogeneous nuclear RNA K-homology (KH) domain, RNA recognition motif (RRM), and the zinc-finger (Znf) domain [110]. In addition, RBPs interact with other RBPs and/or ncRNAs such as miRNAs and lncRNAs via cooperative or competitive interaction [111, 112].

### 3.1. DNA Damage and Oxidative Stress

#### 3.1.1. HuR

HuR is a member of human antigen (Hu) family and governs turnover and translation of target mRNAs involved in the regulation of cell proliferation, growth, survival, and differentiation in response to various stresses [113]. HuR has been reported to have protective roles during DDR and oxidative stress by governing RNA stability and translation of various target mRNAs including *VEGF*, *HIF-1α*, *p53*, *c-myc*, *SIRT1*, and *prothymosin α*. The roles of HuR in the regulation of stress response have been extensively reviewed by others [114, 115]. A recent report has shown that HuR targets and upregulates heme oxygenase 1 (HO1) during oxidative stress [116]. HuR is also implicated in DDR by directly regulating RNA metabolism of p53, WEE1, and non-POU domain-containing octamer-binding protein (NONO, also known as p54NRB) [117, 118]. Esophageal cancer-related gene 2 (ECRG2), a DNA damage-inducible tumor suppressor, can regulate XIAP-mediated cell death by downregulating HuR expression [119].

#### 3.1.2. Heterogeneous Nuclear Ribonucleoproteins

Heterogeneous nuclear ribonucleoproteins (hnRNPs) are nuclear proteins regulating a broad spectrum of RNA metabolism including alternative splicing, translocation, and translation [120]. hnRNP A0 is phosphorylated by MAPK-activated protein kinase 2 (MK2) and stabilizes *GADD45α* mRNA during DDR [121]. In response to UV radiation or hypoxic stress, hnRNP A18 is induced and has protective roles by increasing the expression of UV- or stress-response genes such as *replication protein A* (*RPA2*), *thioredoxin* (*TRX*), or *HIF-1α* [122, 123]. hnRNP A1 is reported to regulate alternative splicing of *hdm2* and UVE-triggered translation of *Apaf-*1 in response to UV exposure [124]. After ionizing radiation, hnRNP C has been found in DNA-damage sites and regulates BRCA gene expression and homologous recombination. Depletion of hnRNP C reduces the abundance of key HR proteins including BRCA1/2, RAD51, and BRIP1 by affecting alternative splicing [125]. hnRNP H/F is reported to increase after DNA damage and to enhance p53 expression by interfering 3′-end processing of *p53* mRNA, thereby regulating apoptosis [126]. hnRNP I (also known as PTB) has been known to increase *HIF-1α*-mediated gene expression by enhancing translation of *HIF-1α* in hypoxia [127].

#### 3.1.3. FUS

FUS (also known as hnRNP P2) binds RNA and single- and double-stranded DNA, and it affects multiple steps of DNA/RNA metabolism. FUS has been observed in sites with laser-induced DNA double-strand breaks (DSBs) and regulates DSB repair [128]. FUS also has an important role in the DDR in neurons by directly interacting with histone deacetylase 1 (HDAC1), and recruitment of FUS and HDAC1 is essential for DDR signaling [129].

#### 3.1.4. T Cell-Restricted Intracellular Antigens

T cell-restricted intracellular antigen-1 (TIA-1) is a member of RNA-binding protein involved in alternative pre-mRNA splicing and mRNA translation. TIA-1 is a component of stress granules (SGs) triggered by hypoxia, ischemia, and anoxia, and it has essential roles in regulating mRNAs involved in oxidative stress and DDR through its associations with other SG components [130]. A recent study has shown that TIA-1 oxidation, mediated by reactive oxygen species (ROS), suppresses SG formation and increases cell death after oxidative stress [131]. TIA-related protein (TIAR) has been reported to increase and regulate neuronal cell death after cerebral ischemic injury [132]. After UVC-induced DNA damage, TIAR is dissociated from C-rich motif-containing mRNAs, including *Apaf-1* mRNA, and enhances their translation [133].

#### 3.1.5. Wig1

Wig1 (also known as ZMAT3) is a transcriptional target gene of p53 and has a zinc-finger domain that binds to double-strand RNA (dsRNA) [134]. Wig1 has been known to stabilize *p53* mRNA by protecting it from deadenylation, thereby enhancing the p53-mediated stress response [135]. Depletion of Wig1 is responsible for increases in cell death and cell-cycle arrest upon DNA damage. Wig1 functions as a survival factor during stress response by regulating FAS and 14-3-3*σ* [136].

### 3.2. Cellular Senescence and Aging

#### 3.2.1. HuR

HuR is implicated in cellular senescence and the aging process based on its involvement in regulating stability and translation of various target mRNAs including *p21*, *p16*, *cyclin A*, *cyclin B1*, *c-fos*, and *SIRT1* [137]. Recent reports have shown that loss of HuR is related to a shorter life span in *Drosophila* as well as to several senescence-associated phenotypes in mouse embryonic fibroblasts (MEF) [138, 139]. The HuR level is downregulated in RS and aging, and its expression is controlled by positive feedback mechanisms [140]. Coactivator-associated arginine methyltransferase 1 (CARM1) has been known as a regulator of HuR by inducing methylation on R217 residue of HuR, and loss of CARM1 downregulates HuR activity in RS [141].

#### 3.2.2. AU-Rich Element RNA-Binding Protein 1

AU-rich element RNA-binding protein 1 **(**AUF1; also known as hnRNP D) includes four alternative spliced isoforms (p37, p40, p42, and p45) containing two RRMs and regulates mRNA stability and turnover. In addition, AUF1 has been shown to affect proliferation, stress response, immune response, and cellular senescence. AUF1 is differentially regulated during aging and cellular senescence [140]. AUF regulates the mRNA stability of *p21* and *p16* in a competitive or cooperative manner with HuR and influences cellular senescence [142, 143]. Pont et al. reported that AUF1-deficient mice exhibit decreased telomerase level and activity, increased DNA damage at telomere ends, enhanced cellular senescence, and rapid premature aging [144].

#### 3.2.3. TIA-1/TIAR

TIA-1 and TIAR regulate alternative splicing, SG formation, and translation of various target genes including *TNFα*, *COX-2*, *c-myc*, *calmodulin 2*, *small nuclear ribonucleoprotein polypeptide F* (*SNRPF*), and *caspase-8* in response to various cellular stresses [145]. It has been shown that TIA-1 is downregulated during RS and aging [140]. TIA-1/TIAR depletion promotes cellular senescence of MEF cells [146]. However, the detailed mechanisms underlying TIA-1/TIAR-mediated regulation of cellular senescence or aging need to be elucidated.

#### 3.2.4. CUGBP1

CUG triplet repeat, RNA-binding protein 1 (CUGBP1) is a member of the CELF/BRUNOL protein family containing two N-terminal RRMs and regulates pre-mRNA alternative splicing, mRNA editing, and translation [147]. CUGBP1 has a role in enhancing p21 expression and regulates cellular senescence [148]. CUGBP1 binds to the 5′-UTR of *p21* and increases translation of *p21* by competing with calreticulin. In senescent cells, increased phosphorylation of CUGBP1 promotes binding to *p21* mRNA.

CUGBP1 has been known to increase with aging in fat tissue and to regulate CCAAT/enhancer-binding protein *β* (C/EBP*β*) expression [149]. CUGBP1 binds to *C/EBPβ* mRNA and enhances its translation, thereby accumulating C/EBP*β*-liver-enriched inhibitory protein (C/EBP*β*-LIP), a dominant inhibitor of differentiation, in fat cells. Augmented expression of CUGBP1 is responsible for the impairment of adipogenesis in aged-fat tissues. In old liver, CUGBP1 phosphorylation at S302 residue by GSK3*β* facilitates the association of CUGBP1 with eukaryotic initiation factor 2 (eIF2) and increases translation of *HDAC1* and *C/EBPβ*, which are responsible for epigenetic regulation of gene expression [150, 151].

#### 3.2.5. Tristetraprolin

Tristetraprolin (TTP) is an ARE-binding protein involved in destabilizing target mRNAs, and its expression is upregulated during cellular senescence and aging [152]. TTP is elevated in B lymphocytes from aged mice compared to the level in cells from young mice, and it destabilizes transcription factor *E47* mRNA [153]. Sanduja et al. have reported that TTP promotes cellular senescence by destabilizing E6-AP ubiquitin ligase mRNA [154]. E6-AP downregulation mediated by TTP results in p53 and hTERT accumulation in cells.

#### 3.2.6. Wig1

Wig1 is also implicated in the regulation of cellular senescence. Kim et al. reported that Wig1 prevents cellular senescence by regulating p21 expression [155]. Wig1 binds to the stem-loop structure near the miRNA-binding site of *p21* mRNA and recruits the RNA-induced silencing complex (RISC) by interacting with Ago2, thereby destabilizing *p21* mRNA. Depletion of Wig1 results in a decrease of miR-mediated *p21* mRNA decay and promotes cellular senescence via p21 upregulation in various cell types.

### 3.3. Age-Related Diseases

#### 3.3.1. Neurodegenerative Diseases

Neuronal cells have their own systems for regulating RNA expression in response to various stimuli via RBPs that are uniquely expressed in neuronal cells. Accumulating evidence indicates that abnormalities in RNA metabolism are a common feature of neurodegeneration [156, 157]. Therefore, mutations or dysregulation of RBPs is widely involved in the pathogenesis of neurodegenerative diseases, including amyotrophic lateral sclerosis, AD, HD, and PD, by governing RNA metabolism [158].

*(1) TDP-43*. TDP-43 was identified as the major component of ubiquitin-positive neuronal inclusion bodies observed in amyotrophic lateral sclerosis (ALS) and frontotemporal lobar degeneration (FTLD) patients [159]. TDP-43 functions as a translational repressor and known to have essential roles in transcriptional regulation and miRNA maturation [160]. Also, TDP-43 regulates axonal transport of RNA granules by interacting with hnRNP A2/B1 [161, 162]. Mutations of TDP-43 genes found in ALS patients are related to delocalization and aggregation of TDP-43. Formation of insoluble aggregates of TDP-43 in the cytoplasm alters interactions between TDP-43 and its target mRNAs having important functions in the brain, thereby indicating the involvement of TDP-43 in ALS/FTLD pathogenesis [163].

*(2) FUS*. Mutations in the gene coding FUS are found in 5% of familial ALS patients and in rare sporadic cases. Like TDP-43, mutations on FUS gene facilitate delocalization and abnormal aggregation of FUS to cytoplasm and affect the alternative splicing of its target genes [164, 165]. Also, FUS mutations are responsible for an increase in DNA damage in ALS patients [129].

*(3) HuD*. HuD (also known as nELAVL or ELAVL4) is expressed in the brain and has been implicated in various aspects of RNA metabolism [166]. HuD functions as a pivotal regulator of neurogenesis, axonal growth, and neuronal function, and dysregulation of HuD results in neuronal defects [167]. HuD has been reported to increase in the brain of AD patients and to stabilize *APP* mRNA, *β-site APP-cleaving enzyme 1* (*BACE1*) mRNA, and BACE1 antisense (BACE1-AS) lncRNAs, thereby facilitating the accumulation of the toxic APP cleavage product A*β* [168].

*(4) FMRP*. Fragile X mental retardation protein (FMRP) is a gene product encoded by fragile X mental retardation 1 (FMR1) and plays essential roles in normal cognitive development and female reproductive function [169]. Mutations on the *FXR1* gene lead to fragile X syndrome (FXS), autism, AD, and PD by dysregulating translation of its target genes [170]. FMRP inhibits *APP* mRNA translation by recruiting *APP* mRNA into P-bodies [171]. FMRP has been shown to decrease in the brain of sporadic AD patients [172].

*(5) hnRNPs*. hnRNP A1 has essential roles in the regulation of pre-mRNA processing, transport, and translation of mRNAs [173]. Loss of hnRNP A1 expression or presences of mutations (D262) are observed in ALS patients [162]. In addition, hnRNP A1 shows a decrease in the AD brain and has been known to regulate alternative splicing of *RAGE* and *APP* mRNAs [174, 175].

hnRNP A2/B1 affects alternative splicing of ALS-associated D-amino acid oxidase, and ALS mutant (hnRNP A2B1 D290V) dysregulates cellular stress responses [162, 176]. hnRNP A2/B1 and hnRNP B1 are also differentially expressed in the AD brain [177].

hnRNP C has been known to stabilize *APP* mRNA or enhance its translation by competing with FMRP, therefore positively regulates APP expression [171]. Borreca et al. reported augmented expression of hnRNP C in the brain of sporadic AD patients [172].

#### 3.3.2. Metabolic Diseases

Metabolic disease is associated with the risk of developing T2DM, obesity, cardiovascular disease (CVD), and coronary heart disease (CHD) [178, 179]. Increasing evidence indicates that dysregulation of RNA metabolism in metabolically active and insulin-sensitive organs, such as the pancreas, liver, muscle, and adipose tissues, is actively implicated in the pathogenesis of metabolic diseases [180].

*(1) HuD*. HuD is also found in the islets of the pancreas and mediates RNA quality control of pancreatic *β* cells [181]. In the pancreatic islets of a T2DM mouse model, HuD expression is downregulated [163]. HuD regulates insulin biosynthesis by associating with 5′-UTR of *insulin2* mRNA and repressing its translation [181]. In addition, HuD regulates autophagosome formation and lipid synthesis via translation regulation of *ATG5* and *INSIG1*, respectively [182, 183]. Moreover, HuD regulates apoptosis of pancreatic *β* cells [184].

*(2) CUGBP1*. CUGBP1 has been reported to regulate insulin resistance and alternative splicing of the insulin [185]. In addition, CUGBP1 negatively regulates insulin secretion by stabilizing phosphodiesterase subtype 3B (PDE3B) [186]. CUGBP1 expression is higher in diabetic heart and pancreas, and single nucleotide polymorphisms (SNPs) on the CUGBP1 locus are associated with obesity [186, 187].

*(3) RBFOX2*. RBFOX2 (also known as RBM9) is a RNA-binding protein and a homolog of *C. elegans* Fox-1 and regulates alternative splicing by directly binding to the consensus (U)GCAUG motif in the target pre-mRNAs [188]. A recent study by Nutter et al. showed that 73% of transcripts misspliced in diabetic hearts have RBFOX2-binding sites, and a dominant negative form of RBFOX2 (DN-RBFOX2) was found in diabetic hearts [189]. DN-RBFOX2 precedes diabetic cardiac complications, as well as delays intracellular calcium transients in cardiomyocytes by blocking RBFOX2-mediated alternative splicing.

*(4) IGF2BP2/IMP2*. Insulin-like growth factor 2 mRNA-binding protein 2 (IGF2BP2/IMP2) belongs to *IGF2* mRNA-binding protein (IMP) family and is known to regulate *IGF2* translation by interacting with the 5′-UTR of *IGF2* mRNA [190]. Genome-wide association studies have shown that the human IGF2BP/IMP2 gene contains SNPs associated with T2DM [191, 192]. Dai et al. demonstrated that mice lacking IGF2BP2/IMP2 resist diet-induced obesity and have improved glucose tolerance, insulin sensitivity, and longer lifespan through the increased translation of UCP1 or mitochondrial components [193].

**Figure 3:**
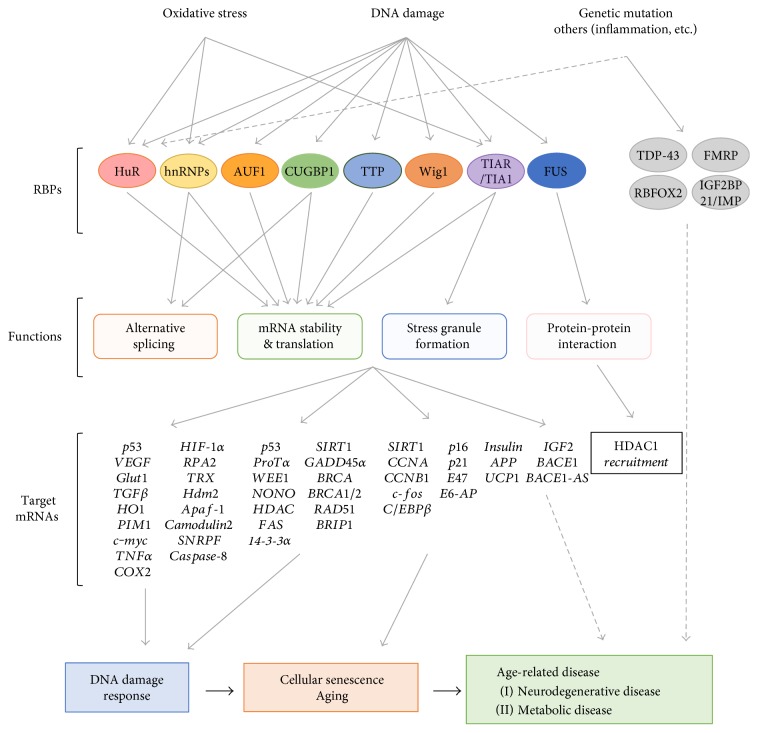
Representative RBPs involved in DNA damage and oxidative stress, cellular senescence, and age-related diseases.

Representative RBPs involved in DNA damage and oxidative stress, cellular senescence, and age-related diseases are shown in Figure 3.

## 4. Conclusion

Increasing evidence indicates that ncRNAs and RBPs are essential regulators of various cellular processes, and dysregulation of these RNA regulators is implicated in the pathogenesis of several diseases including neurodegenerative diseases, metabolic diseases, and cancer. In this review, we tried to discuss the regulatory lncRNAs and RBPs that are involved in stress response, cellular senescence, and the pathogenesis of age-related diseases including neurodegenerative diseases, metabolic diseases, immune response, and muscle dysfunction (Tables 1 and 2). We have limited our discussion to lncRNAs and RBPs because miRNAs have been intensively reviewed by others [194–196]. Although the list of reviewed lncRNAs and RBPs is extensive, additional RNA regulators are certainly going to be uncovered in future studies of stress-related responses and age-related diseases.

The results of studies undertaken to uncover the roles of lncRNAs and RBPs during stress response, cellular senescence, and the pathogenesis of age-related diseases are prompting several questions for immediate consideration. For example, what are the molecular targets of lncRNAs and RBPs? What signaling pathways control the expression and function of lncRNAs and RBPs during the stress response or in the pathogenesis of age-related diseases? How do they contribute to the stress response and cellular senescence? Do lncRNAs and RBPs interplay in order to fine tune RNA metabolism? How are RNA regulators including lncRNAs, miRNAs, and RBPs differentially expressed in age-related diseases? As we begin to consider these questions, the importance of functional networking between RBPs and ncRNAs is coming to the forefront [197–199].

A deeper and more comprehensive knowledge of the fine mechanisms involving lncRNAs and RBPs in the regulation of RNA metabolism is warranted because regulatory lncRNAs and RBPs are promising novel targets for intervention in physiopathologies with underlying deficiencies in stress response, cellular senescence, and the aging process.

## Figures and Tables

**Table 1:** A list of lncRNAs involved in DNA damage response, oxidative stress, cellular senescence, and age-related diseases.

LncRNAs	Functions	References
*DNA damage response*		
*LincRNA-p21*	Represses gene expression with hnRNP K	[20–22]
*LincRNA-RoR*	Suppresses p53 translation with hnRNP I and inhibits p53-mediated cell-cycle arrest and apoptosis	[23–25]
*Pint*	Connects p53 activation with epigenetic silencing by PRC2	[20, 26]
*PANDA*	Regulates proapoptotic genes with NF-YA	[27–29]
*LncRNA-JADE*	Connects the DNA damage response to histone H4 acetylation	[30]
*Oxidative stress*		
*H19*	Upregulated by oxidative stress	[31–34]
*ANRIL*	Represses the expression of INK4A-ARF-INK4B	[35]
*LncRNA-LET*	Degrades NF90 via ubiquitin-proteasome pathway	[36]
*LINK-A*	Regulates the stabilization of HIF-1*α*	[37]
*Cellular senescence*		
*7SL*	Promotes cell growth via suppression of p53	[38, 39]
*HOTAIR*	Represses transcription of HOXD with PRC2	[40–42]
*UCA1*	Negative correlation between p27 and UCA in breast cancer tissue	[43–45]
*LincRNA-p21*	Influences the p53 tumor suppressor pathway by regulating p53-mediated p21 expression	[46]
*ANRIL*	Regulates CDKN2A/B by epigenetic mechanisms	[47–52]
*ANRASSF1*	Represses the expression of RASSF1A	[53, 54]
*PANDA*	Interacts with PRC1, PRC2, and NF-YA and represses the transcription of senescence-promoting genes	[55, 56]
*FAL1*	Oncogenic activity of FAL1 is repression of p21	[57, 58]
*MIR31HG*	Interacts with both INK3A and PcG proteins and represses INK4A	[59]
*SALNR*	Regulates NF90 activity	[60]
*VAD*	Regulates chromatin structure and increases the expression of INK4	[61]
*Neurodegenerative diseases*		
*BC200*	Upregulation of BC200 related to the severity of AD	[63, 64]
*BACE1-AS*	Regulates BACE1 mRNA and generates A*β* 1–42	[65, 66]
*NDM29*	Induces APP and increases A*β* secretion	[67, 68]
*17A*	Enhances A*β* secretion by impairing GABA-B signaling	[69, 71]
*AS Uchl1*	Induces Uchl1 expression by increasing its translation	[73]
*naPINK1*	Regulates the stabilization of svPINK1 expression	[71, 74]
*TUG1*	Downstream target of p53 and regulates cell-cycle genes	[76–79]
*MEG3*	Epigenetically regulates chromatin in HD	[16, 78]
*HTTAS-V1*	Overexpression of HTTAS-V1 reduces HTT transcripts	[80]
*Immune response*		
*THRIL*	Regulates TNF*α* expression and is associated with childhood acute inflammatory diseases	[82]
*Lnc-DC*	Exclusively expressed in dendritic cells and regulates DC differentiation	[83]
*Lnc-IL7R*	Diminishes LPS-induced inflammatory response	[84]
*LincRNA-EPS*	Regulated in macrophages to control the expression of immune response genes	[85]
*Diabetes*		
*RNCR3*	Regulates retinal endothelial cell function via RNCR3/KLF2/miR-185-5p	[87, 88]
*MEG3*	Downregulates MEG3 in the retinas of STZ-induced diabetic mice	[89, 90]
*HI-LNC25*	Regulates *β* cell differentiation and maturation	[91, 92]
*Muscle dysfunction*		
*SRA*	Enhances the activity of nuclear receptors and regulates differentiation of MyoD	[94, 95]
*MUNC*	Facilitates the function of MyoD in skeletal myogenesis	[96]
*Linc-RAM*	Promotes assembly of MyoD-Baf60-Brg1 complex and increases the transcription of myogenic differentiation genes	[97]
*Atherosclerosis*		
*SENCR*	Impedes migration and proliferation of smooth muscle cells by regulating FOXO1 and TRPC6 expression	[101–103]
*Cataracts*		
*LncRNA-MIAT*	Upregulated in patients with cataracts and involved in the maintenance of LECs	[106]

**Table 2:** A list of RBPs involved in DNA damage response, oxidative stress, cellular senescence, and age-related diseases.

RBPs	Functions	References
*DNA damage response and oxidative stress*		
HuR	Protection roles in oxidative stress and DNA damage by regulating RNA metabolism (reviewed in 114, 115) Regulates HO1, WEE1, and NONO expression during stress response	[114–119]
hnRNP A0	Phosphorylation of hnRNP A0 by MK2 promotes *GADD45α* mRNA stabilization	[121]
hnRNP A18	Increases gene expression involved in stress-response	[122, 123]
hnRNP A1	Involved in alternative splicing of *hdm2* and *Apaf-1* translation	[124]
hnRNP C	Regulates BRCA gene expression and homologous recombination after ionizing irradiation	[125]
hnRNP H/F	Increased in DNA damage response and upregulates p53 expression	[126]
hnRNP I	Enhances translation of *HIF-1α* in hypoxia	[127]
FUS	Interacts with HDAC1 and regulates DNA damage response	[129]
TIA-1/TIAR	TIA-1/TIAR are involved in SG formation after stress response and decrease *HIF-1α* translation TIA-1 oxidation by ROS suppresses SG formation and increases cell death TIAR increases *Apaf-1* translation after UVC-induced DNA damage	[130, 131, 133]
Wig1	Stabilizes *p53* mRNA and enhancing p53-mediated stress response	[136]
*Cellular senescence and aging*		
HuR	HuR loss is related to shorter life span and enhanced senescence-associated phenotypes (reviewed in 137) CARM1 downregulates HuR activity in replicative senescence	[137–139, 141]
AUF1	Involved in cellular senescence by regulating mRNA stability of *p21* and *p16*, and AUF1 KO mice show enhanced cellular senescence and rapid premature aging	[142–144]
TIA-1/TIAR	Down-regulated in cellular senescence and TIA-1/TIAR depletion promotes cellular senescence of MEF cells	[140, 146]
CUGBP1	CUGBP1 phosphorylation promotes the binding to *p21* mRNA in senescent cells Regulates C/EBP*β* and HDAC1 in the liver and fat of old mice	[148, 150, 151]
TTP	Upregulated in senescent cells and contributes to p53 accumulation by destabilizing *E6-AP* mRNA	[140, 154]
Wig1	Prevents premature senescence by destabilizing *p21* mRNA	[155]
*Neurodegenerative diseases*		
TDP-43	Functions as a translational repressor Regulates axonal transport of RNA granules by interacting with hnRNP A2/B1 Mutants form of TDP-43 found in ALS patients are prone to aggregation	[160–162]
FUS	Interacts with DNA/RNA and regulates DNA/RNA metabolism Mutation found in ALS patients are related to abnormal aggregation of FUS in cytoplasm and dysregulation of alternative splicing	[164]
HuD	Has pivotal roles in neurogenesis, axonal growth, and neuronal functions Upregulated in the brain of AD patients and promotes A*β* accumulation	[166, 168]
FMRP	Mutations on FXP1 gene are linked to FXS, AD, and PD by dysregulation translation of target genes Downregulated in the brain of sporadic AD patients and regulates *APP* translation	[170–172]
hnRNP A1	Loss of hnRNP A1 or mutations on D262 residue is found in the ALS patients Downregulated in the brain of AD patients and affects to splicing of *RAGE* and *APP* mRNAs	[174, 175]
hnRNP A2/B1	Mutation on D290 residue dysregulates cellular stress response in ALS Differentially expressed in the brain of AD and affects alternative splicing	[176, 177]
hnRNP C	Upregulated in the brain of AD patients Stabilizes *APP* mRNA and enhances translation of *APP*	[171, 172]
*Metabolic diseases*		
HuD	Downregulated in the pancreas of T2DM Regulates insulin biosynthesis, autophagosome formation, lipid synthesis, and apoptosis in pancreatic *β* cells	[181–184]
CUGBP1	Upregulated in the diabetic hearts and the pancreas and regulates insulin secretion and insulin resistance Obesity-related SNPs on CUGBP1 influence alternative splicing, translation, and turnover of target mRNAs	[185–187]
RBFOX2	Plays essential roles in alternative splicing In diabetic hearts, majority of misspliced transcripts have RBFOX2-binding sites	[188, 189]
IGF2BP2/IMP	SNPs on IGF2BP2/IMP2 genes are associated to T2DM IMP2 KO mice show better glucose tolerance, insulin sensitivity, and longer lifespan	[191–193]
